# Supplementary figures and images for: Dopamine promotes instrumental motivation, but reduces reward-related vigour
Source: eLife. 2020 Oct 1;9:e58321. doi: 10.7554/eLife.58321 (PMC7599069; doi:10.7554/eLife.58321)

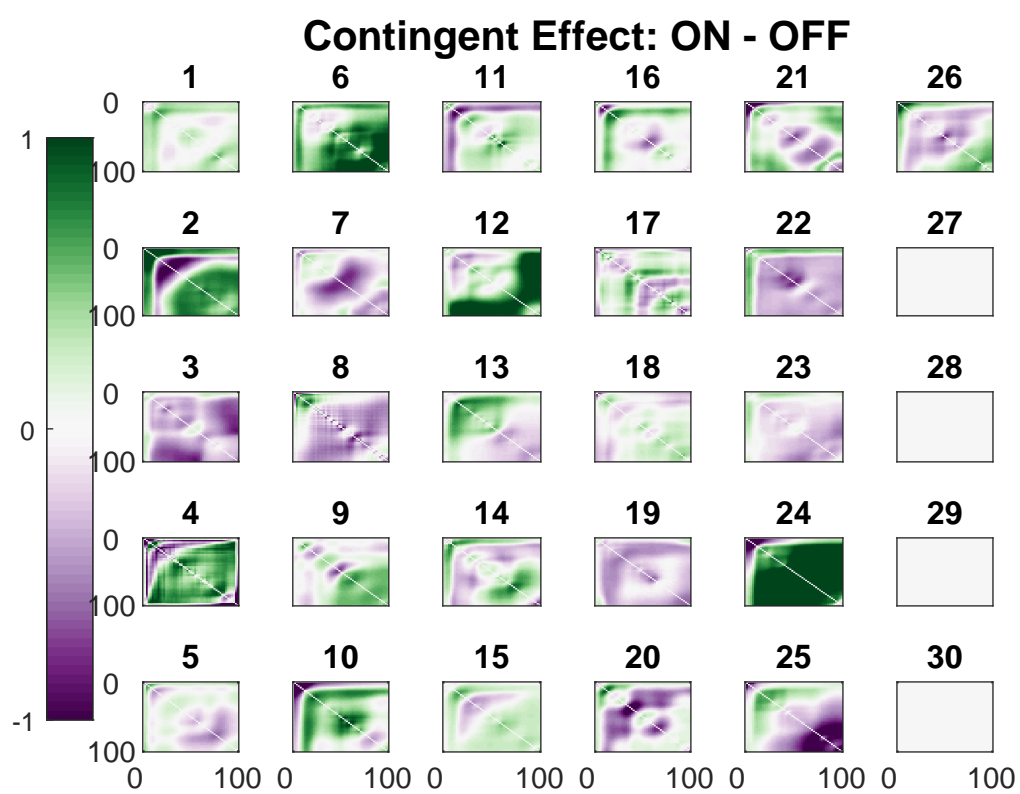

### Contingent Effect: ON

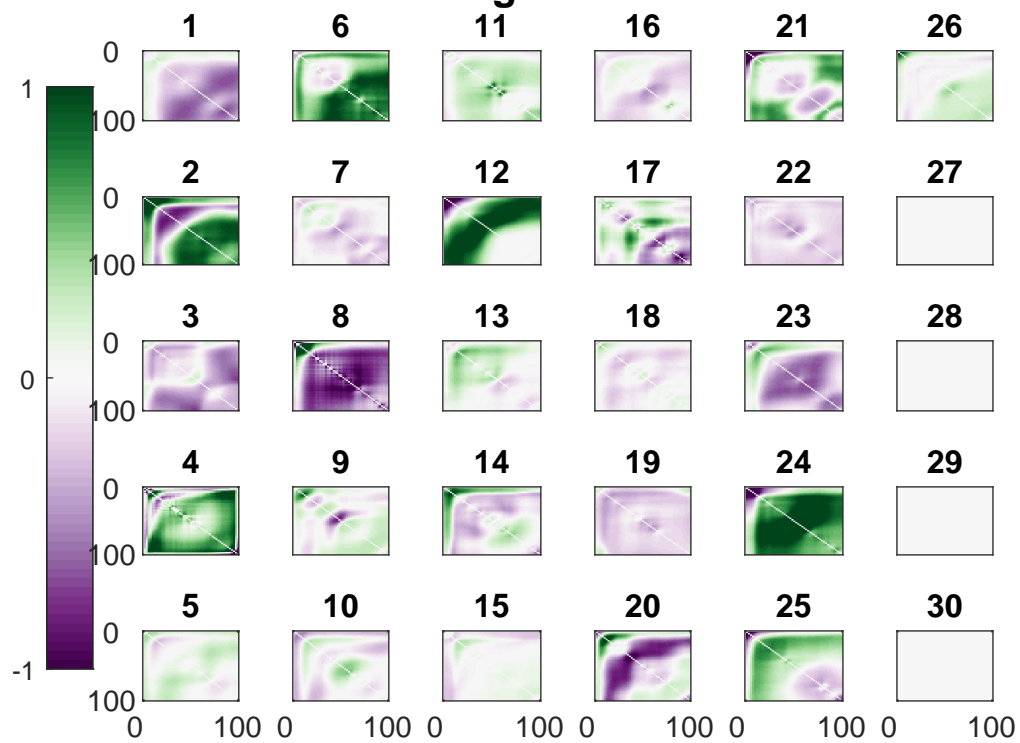

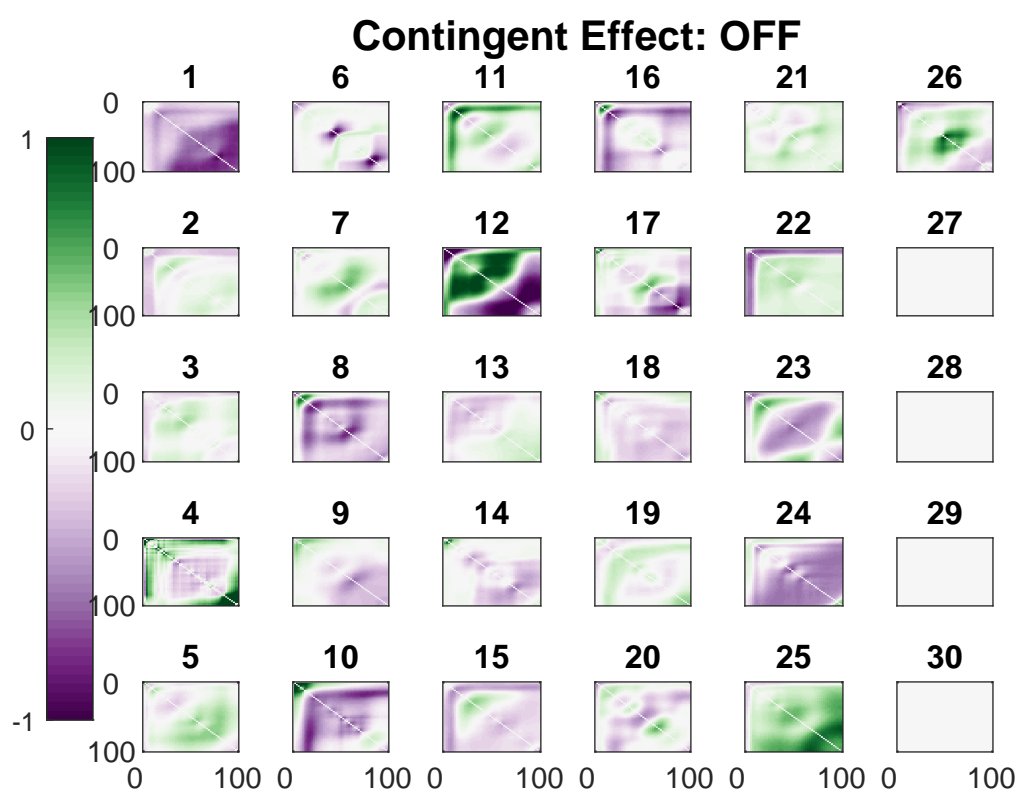

### Contingent Effect: HC

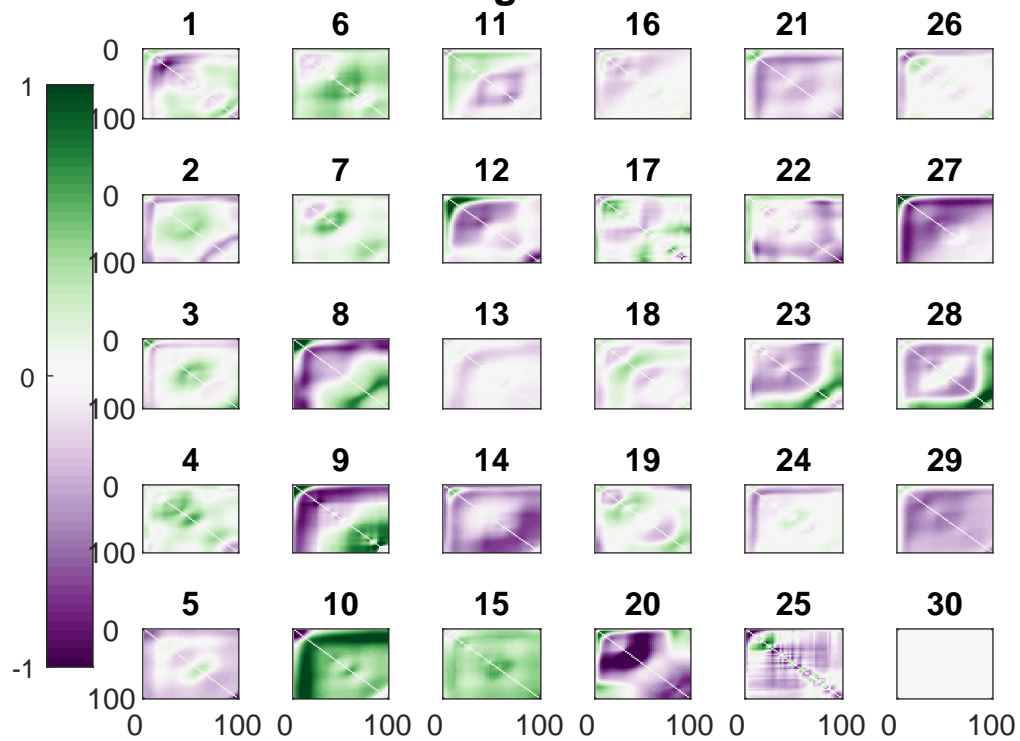

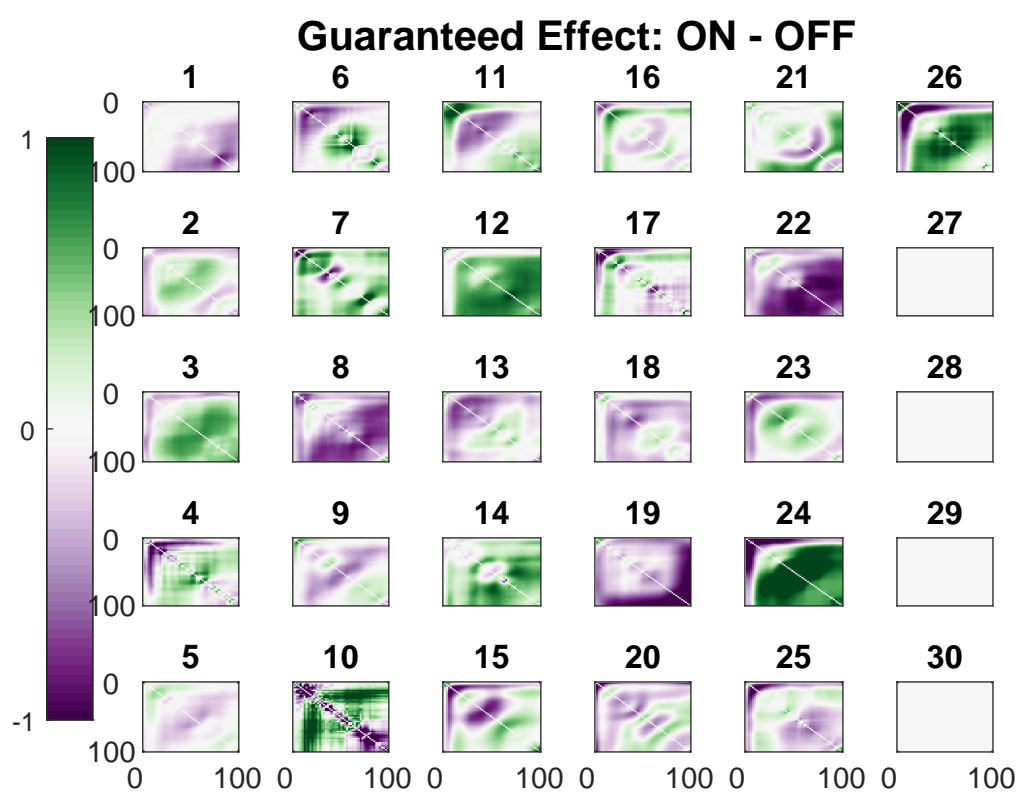

Guaranteed Effect: ON

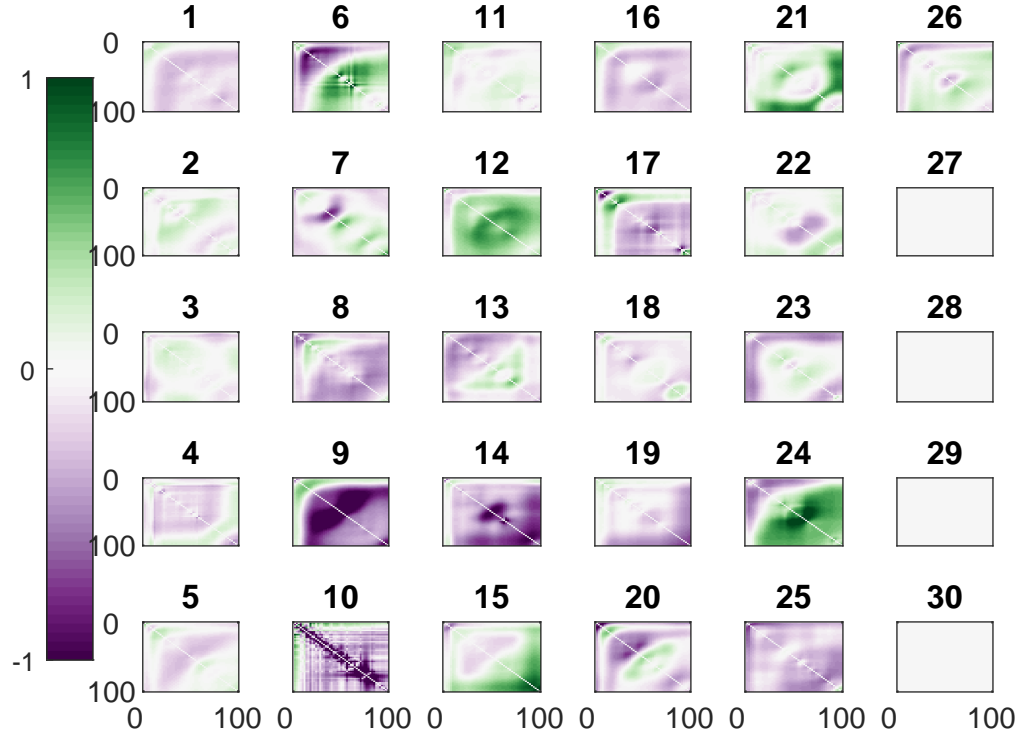

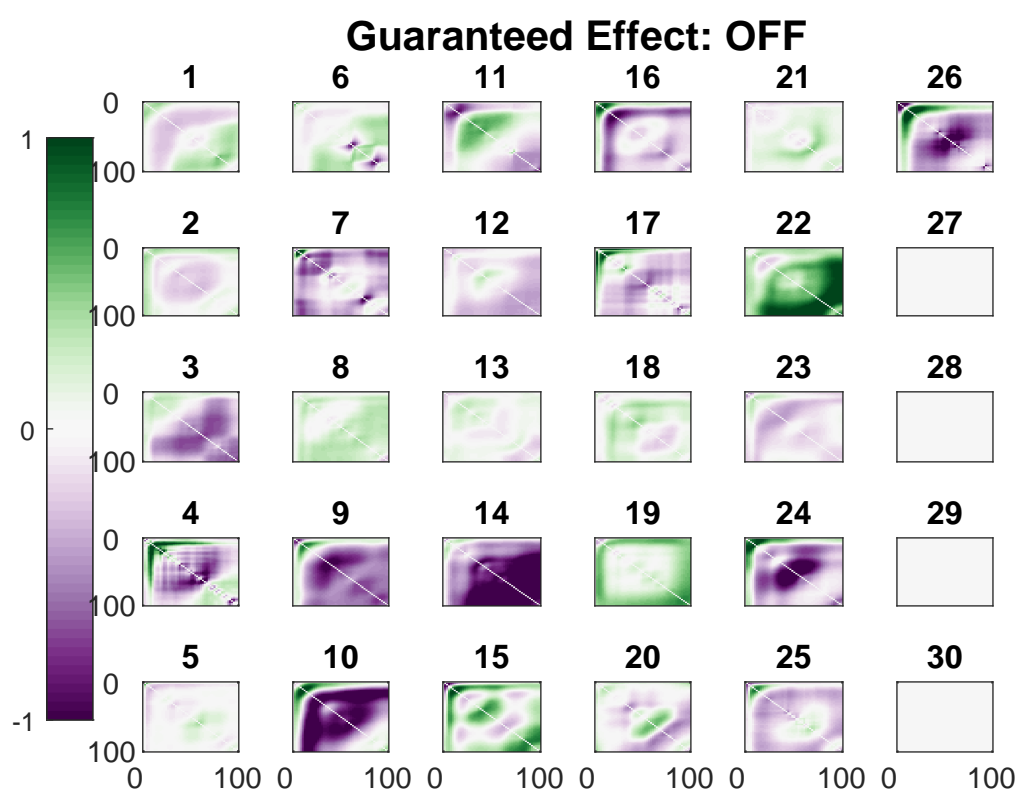

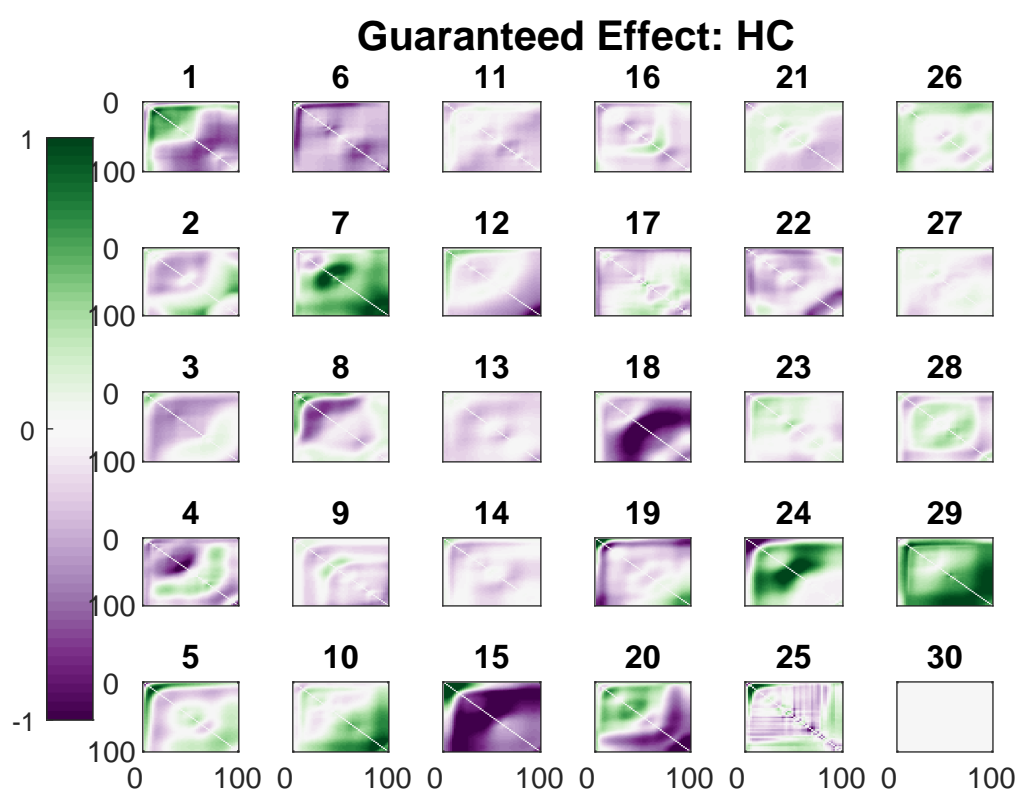

Supplement: Figure 4—source data 2. — Individual data showing each participant’s mean effects of contingent (Performance – Random) and guaranteed (10 p – 0 p) rewards on (Fisher transformed) eye-position autocorrelation matrices. Blank matrices for PD patients reflect excluded participants. [file elife-58321-fig4-data2.pdf]
