# Supplementary material for: Dopamine promotes instrumental motivation, but reduces reward-related vigour
Source: eLife. 2020 Oct 1;9:e58321. doi: 10.7554/eLife.58321 (PMC7599069; doi:10.7554/eLife.58321)
Supplement: Supplementary file 1. [file elife-58321-supp1.docx]

Supplementary File 1 – Statistics for main saccade measures

# A.

Table A. Statistics for two-way ANOVA on peak velocity residuals for each PD condition, separately. PD ON had effects of motivation, contingency and a trend interaction of these (as motivation increased peak velocity only for the contingent condition). PD OFF had no overall effect of contingency (as peak velocity was higher for guaranteed conditions) and a borderline significant motivation*contingency interaction (as 10p had the highest velocity). * = p < .05, ** = p < .01.

| Group | Effect | F | p | $\boldsymbol{\eta}_{\boldsymbol{p}}^{\boldsymbol{2}}$ |
| --- | --- | --- | --- | --- |
| PD ON (*df* = 1, 100) | Motivation | 7.8283 | **.0062 | .0726 |
|  | Contingency | 7.1289 | **.0089 | .0665 |
|  | Motivation * Contingency | 5.8942 | *.0170 | .0557 |
| PD OFF (*df* = 1, 100) | Motivation | 2.9575 | .0886 | .0287 |
|  | Contingency | 4.4168 | *.0381 | .0423 |
|  | Motivation * Contingency | 3.9330 | .0501 | .0378 |


# B

Table B. Statistics for HC vs PD ON behavioural analyses. PD ON did not differ in velocity residuals from HC. There was a significant motivation*contingency interaction for endpoint variability, as both HC and PD ON showed greater variability after contingent rewards, but less variability after expected rewards. HC had greater amplitudes, quicker RTs and smaller variabilities than PD ON. * = p < .05, *** = p < .001

| Measure | Effect | F (*df* = 1, 220) | p | $\boldsymbol{\eta}_{\boldsymbol{p}}^{\boldsymbol{2}}$ |
| --- | --- | --- | --- | --- |
| Velocity residuals | Motivation | 4.1837 | *.0421 | .0194 |
|  | Contingency | 2.8162 | .0948 | .0131 |
|  | Group | 0.0008 | .9769 | .0000 |
|  | Motivation * Contingency | 3.1877 | .0756 | .0148 |
|  | Motivation * Group | 0.1677 | .6826 | .0008 |
|  | Contingency * Group | 0.4415 | .5071 | .0021 |
|  | Contingency * Motivation * Group | 0.1188 | .7306 | .0006 |
| Amplitude | Motivation | 2.5358 | .1128 | .0118 |
|  | Contingency | 0.0062 | .9371 | .0000 |
|  | Group | 85.0115 | ***<.001 | .2862 |
|  | Motivation * Contingency | 0.3183 | .5732 | .0015 |
|  | Motivation * Group | 0.7524 | .3867 | .0035 |
|  | Contingency * Group | 0.0239 | .8773 | .0001 |
|  | Contingency * Motivation * Group | 0.0012 | .9726 | .0000 |
| Saccade RT | Motivation | 1.6851 | .1957 | .0079 |
|  | Contingency | 0.8771 | .3501 | .0041 |
|  | Group | 23.0563 | ***<.001 | .0981 |
|  | Motivation * Contingency | 0.4339 | .5108 | .0020 |
|  | Motivation * Group | 0.0645 | .7998 | .0003 |
|  | Contingency * Group | 0.0000 | .9974 | .0000 |
|  | Contingency * Motivation * Group | 0.0989 | .7535 | .0005 |
| End-point Variability | Motivation | 0.3487 | .5555 | .0016 |
|  | Contingency | 0.4345 | .5105 | .0020 |
|  | Group | 55.8485 | ***<.001 | .2085 |
|  | Motivation * Contingency | 4.9883 | * .0266 | .0230 |
|  | Motivation * Group | 0.0025 | .9601 | .0000 |
|  | Contingency * Group | 0.0138 | .9066 | .0001 |
|  | Contingency * Motivation * Group | 0.1040 | .7474 | .0005 |
| Raw Peak Velocity | Motivation | 1.1524 | .2843 | .0054 |
|  | Contingency | 0.2321 | .6304 | .0011 |
|  | Group | 3.9372 | *.0485 | .0182 |
|  | Motivation * Contingency | 0.2280 | .6335 | .0011 |
|  | Motivation * Group | 0.1300 | .7188 | .0006 |
|  | Contingency * Group | 0.0451 | .8319 | .0002 |
|  | Contingency * Motivation * Group | 0.0196 | .8888 | .0001 |

# C

Table C. Statistics for HC vs PD OFF behavioural analyses. HC did not significantly differ from PD OFF in velocity residuals, but HC had greater amplitudes, quicker RTs and lower variability than PD OFF. * = p < .05, *** = p < .001

| Measure | Effect | F (*df* = 1, 220) | p | $\boldsymbol{\eta}_{\boldsymbol{p}}^{\boldsymbol{2}}$ |
| --- | --- | --- | --- | --- |
| Velocity residuals | Motivation | 2.7173 | .1007 | .0127 |
|  | Contingency | 0.3062 | .5806 | .0014 |
|  | Group | 0.0016 | .9678 | .0000 |
|  | Motivation * Contingency | 0.0826 | .7741 | .0004 |
|  | Motivation * Group | 0.0038 | .9508 | .0000 |
|  | Contingency * Group | 2.3610 | .1259 | .0110 |
|  | Contingency * Motivation * Group | 2.8383 | .0935 | .0132 |
| Amplitude | Motivation | 1.3011 | .2553 | .0061 |
|  | Contingency | 1.8546 | .1747 | .0087 |
|  | Group | 89.4129 | ***<.001 | .2966 |
|  | Motivation * Contingency | 0.5945 | .4415 | .0028 |
|  | Motivation * Group | 0.1489 | .6999 | .0007 |
|  | Contingency * Group | 1.6466 | .2008 | .0077 |
|  | Contingency * Motivation * Group | 0.0482 | .8264 | .0002 |
| Saccade RT | Motivation | 1.3548 | .2458 | .0063 |
|  | Contingency | 2.5505 | .1117 | .0119 |
|  | Group | 30.5031 | ***<.001 | .1258 |
|  | Motivation * Contingency | 0.6381 | .4253 | .0030 |
|  | Motivation * Group | 0.0041 | .9490 | .0000 |
|  | Contingency * Group | 0.3769 | .5399 | .0018 |
|  | Contingency * Motivation * Group | 0.0512 | .8212 | .0002 |
| End-point Variability | Motivation | 2.6523 | .1049 | .0124 |
|  | Contingency | 3.0066 | .0844 | .0140 |
|  | Group | 62.8641 | ***<.001 | .2287 |
|  | Motivation * Contingency | 2.6619 | .1043 | .0124 |
|  | Motivation * Group | 1.0998 | .2955 | .0052 |
|  | Contingency * Group | 1.5467 | .2150 | .0072 |
|  | Contingency * Motivation * Group | 0.0096 | .9219 | .0000 |
| Raw Peak Velocity | Motivation | 1.1853 | .2775 | .0056 |
|  | Contingency | 0.0141 | .9055 | .0001 |
|  | Group | 1.7072 | .1928 | .0080 |
|  | Motivation * Contingency | 0.1301 | .7186 | .0006 |
|  | Motivation * Group | 0.0748 | .7848 | .0004 |
|  | Contingency * Group | 0.0357 | .8502 | .0002 |
|  | Contingency * Motivation * Group | 0.5577 | .4560 | .0026 |
