# Supplementary material for: Dopamine promotes instrumental motivation, but reduces reward-related vigour
Source: eLife. 2020 Oct 1;9:e58321. doi: 10.7554/eLife.58321 (PMC7599069; doi:10.7554/eLife.58321)
Supplement: Supplementary file 2. [file elife-58321-supp2.docx]

Supplementary File 2 – Statistics for Pupil Dilatation

# A

*Table A.* Pupil dilatation Window-of-interest statistics for PD ON vs OFF. A repeated-measures ANOVA comparing PD ON vs OFF on the mean pupil dilatation (from baseline) in the window-of-interest (1000-1400ms after the cue). There were no significant effects or interactions.

| Effect | F (*df* = 1, 200) | p | $\boldsymbol{\eta}_{\boldsymbol{p}}^{\boldsymbol{2}}$ |
| --- | --- | --- | --- |
| Motivation | 0.3521 | .5536 | .0018 |
| Contingency | 0.2626 | .6089 | .0013 |
| Drug | 2.3098 | .1301 | .0114 |
| Motivation * Contingency | 0.4529 | .5017 | .0023 |
| Motivation * Drug | 0.1105 | .7399 | .0006 |
| Contingency * Drug | 0.1010 | .7509 | .0005 |
| Motivation * Contingency * Drug | 0.2006 | .6547 | .0010 |

# B

*Table B.* Pupil dilatation Window-of-interest statistics of PD ON vs HC. A repeated-measures ANOVA comparing PD ON vs HC on the mean pupil dilatation (from baseline) in the window-of-interest (1000-1400ms after the cue). HC had smaller dilations than PD ON, but there were no effects of motivations or interactions. *** = p < .001

| Effect | F (*df* = 1, 212) | p | $\eta_{p}^{2}$ |
| --- | --- | --- | --- |
| Motivation | 0.2789 | .5980 | .0013 |
| Contingency | 0.0770 | .7817 | .0004 |
| Group | 17.5954 | ***<.0001 | .0766 |
| Motivation * Contingency | 0.1513 | .6977 | .0007 |
| Motivation * Group | 0.0004 | .9845 | .0000 |
| Contingency * Group | 0.0457 | .8310 | .0002 |
| Motivation * Contingency * Group | 0.0750 | .7844 | .0004 |

# C

*Table C.* Pupil dilatation Window-of-interest statistics of PD OFF vs HC. A repeated-measures ANOVA comparing PD OFF vs HC on the mean pupil dilatation (from baseline) in the window-of-interest (1000-1400ms after the cue). HC had smaller dilations than PD OFF, but there were no effects of motivations or interactions. *** = p < .001

| Effect | F (*df* = 1, 212) | p | $\eta_{p}^{2}$ |
| --- | --- | --- | --- |
| Motivation | 0.1468 | .7020 | .0007 |
| Contingency | 0.0107 | .9178 | .0001 |
| Group | 18.0533 | ***<.0001 | .0785 |
| Motivation * Contingency | 0.0206 | .8861 | .0001 |
| Motivation * Group | 0.0419 | .8379 | .0002 |
| Contingency * Group | 0.0009 | .9763 | .0000 |
| Motivation * Contingency * Group | 0.0001 | .9917 | .0000 |
