# Supplementary material for: Dopamine promotes instrumental motivation, but reduces reward-related vigour
Source: eLife. 2020 Oct 1;9:e58321. doi: 10.7554/eLife.58321 (PMC7599069; doi:10.7554/eLife.58321)
Supplement: Supplementary file 3. [file elife-58321-supp3.docx]

Supplementary File 3 – Statistics for fixation period

# A

*Table A. Repeated-measures ANOVA on microsaccades during fixation*. A repeated-measures ANOVA comparing PD ON vs OFF on the mean number of microsaccades during the 1400ms fixation period. PD OFF had significantly more microsaccades during fixation, but there were no other significant effects or interactions. * = p < .05.

| Effect | F (*df* = 1, 201) | p | $\boldsymbol{\eta}_{\boldsymbol{p}}^{\boldsymbol{2}}$ |
| --- | --- | --- | --- |
| Motivation | 0.0019 | .9654 | .0000 |
| Contingency | 0.6914 | .4067 | .0034 |
| Drug | 5.0451 | *.0258 | .0245 |
| Motivation * Contingency | 0.7205 | .3970 | .0036 |
| Motivation * Drug | 0.1169 | .7327 | .0006 |
| Contingency * Drug | 1.8489 | .1754 | .0091 |
| Motivation * Contingency * Drug | 0.1686 | .6818 | .0008 |

# B

*Table B. Repeated-measures ANOVA on ocular drift speed during fixation*. A repeated-measures ANOVA comparing PD ON vs OFF on the mean ocular drift speed during the 1400ms fixation period. PD OFF had significantly more microsaccades during fixation, but there were no other significant effects or interactions. * = p < .05.

| Effect | F (*df* = 1, 201) | p | $\boldsymbol{\eta}_{\boldsymbol{p}}^{\boldsymbol{2}}$ |
| --- | --- | --- | --- |
| Motivation | 0.9516 | .3304 | .0044 |
| Contingency | 0.1621 | .6876 | .0007 |
| Drug | 5.4327 | *.0207 | .0245 |
| Motivation * Contingency | 0.0390 | .8435 | .0002 |
| Motivation * Drug | 0.0192 | .8898 | .0001 |
| Contingency * Drug | 0.0001 | .9917 | .0000 |
| Motivation * Contingency * Drug | 0.0194 | .8894 | .0001 |
