# Supplementary material for: Dopamine promotes instrumental motivation, but reduces reward-related vigour
Source: eLife. 2020 Oct 1;9:e58321. doi: 10.7554/eLife.58321 (PMC7599069; doi:10.7554/eLife.58321)
Supplement: Supplementary file 4. [file elife-58321-supp4.docx]

Supplementary File 4 – Correlations of questionnaires with motivational effects

# A

Table A. No correlations between apathy or depression and vigour. The outputs from Pearson’s correlations between apathy and depression questionnaires and the contingent and guaranteed motivational effects on residual peak velocity in PD ON and OFF separately. No correlations were significant.

| Questionnaires | Effect | PD ON | PD OFF |
| --- | --- | --- | --- |
| AMI | Contingent | *ρ* = -.3312, p = .0984 | *ρ* = .0429, p = .8352 |
|  | Guaranteed | *ρ* = -.0439, p = .8313 | *ρ* = .1112, p = .5887 |
| HADS | Contingent | *ρ* = .0570, p = .7814 | *ρ* = -.3261, p = .1199 |
|  | Guaranteed | *ρ* = -.2577, p = .2240 | *ρ* = -.0355, p = .8692 |
| BDI-II | Contingent | *ρ* = -.0166, p = .9445 | *ρ* = -.1763, p = .4573 |
|  | Guaranteed | *ρ* = -.1838, p = .4379 | *ρ* = -.0605, p = .7999 |
