# Supplementary material for: Dopamine promotes instrumental motivation, but reduces reward-related vigour
Source: eLife. 2020 Oct 1;9:e58321. doi: 10.7554/eLife.58321 (PMC7599069; doi:10.7554/eLife.58321)
Supplement: Transparent reporting form [file elife-58321-transrepform.docx]

***eLife’s* transparent reporting form**

We encourage authors to provide detailed information *within their submission* to facilitate the interpretation and replication of experiments. Authors can upload supporting documentation to indicate the use of appropriate reporting guidelines for health-related research (see [EQUATOR Network](http://www.equator-network.org/%20)), life science research (see the [BioSharing Information Resource](https://biosharing.org/)), or the [ARRIVE guidelines](http://www.plosbiology.org/article/info:doi/10.1371/journal.pbio.1000412) for reporting work involving animal research. Where applicable, authors should refer to any relevant reporting standards documents in this form.

If you have any questions, please consult our Journal Policies and/or contact us: [editorial@elifesciences.org](mailto:editorial@elifesciences.org).

**Sample-size estimation**

- You should state whether an appropriate sample size was computed when the study was being designed
- You should state the statistical method of sample size computation and any required assumptions
- If no explicit power analysis was used, you should describe how you decided what sample (replicate) size (number) to use

Please outline where this information can be found within the submission (e.g., sections or figure legends), or explain why this information doesn’t apply to your submission:

From the Methods/Participants section:

We recruited 30 participants in each group as this was the sample size used in previous experiments with this task and yielded robust effects (Manohar et al., 2017). Sensitivity power calculations showed this would detect effect sizes above 0.46 (Faul, Erdfelder, Buchner, & Lang, 2009) (α = .05, power = .8, sample size = 30), although as we only included 26 PD in the analysis, this effect size rose to 0.5.

**Replicates**

- You should report how often each experiment was performed
- You should include a definition of biological versus technical replication
- The data obtained should be provided and sufficient information should be provided to indicate the number of independent biological and/or technical replicates
- If you encountered any outliers, you should describe how these were handled
- Criteria for exclusion/inclusion of data should be clearly stated
- High-throughput sequence data should be uploaded before submission, with a private link for reviewers provided (these are available from both GEO and ArrayExpress)

Please outline where this information can be found within the submission (e.g., sections or figure legends), or explain why this information doesn’t apply to your submission:

Each PD patient performed the task twice, once ON and once OFF medication. Healthy Controls performed it once.

Two PD patients withdrew before completing both sessions and were excluded.

Two PD patients and one Healthy Control did not pass our data inclusion criteria (>10 saccades in each condition which have amplitude > 1°, velocity of 80-2500°s^-1^, response times of 180-580ms), leaving 26 PD and 29 HC for all data and analyses. This is stated in the Methods\Analysis section

**Statistical reporting**

- Statistical analysis methods should be described and justified
- Raw data should be presented in figures whenever informative to do so (typically when N per group is less than 10)
- For each experiment, you should identify the statistical tests used, exact values of N, definitions of center, methods of multiple test correction, and dispersion and precision measures (e.g., mean, median, SD, SEM, confidence intervals; and, for the major substantive results, a measure of effect size (e.g., Pearson's r, Cohen's d)
- Report exact p-values wherever possible alongside the summary statistics and 95% confidence intervals. These should be reported for all key questions and not only when the p-value is less than 0.05.

Please outline where this information can be found within the submission (e.g., sections or figure legends), or explain why this information doesn’t apply to your submission:

The Methods\Analysis states we used repeated measures ANOVA for the main comparisons, and for the time-course analyses we used cluster-wise permutation tests to control the family-wise error rate at .05.

N = 26 PD, and 29 HC throughout, which is stated in the methods.

All figures show mean and SEM (stated in legends).

We provide the main test ANOVA outputs (including F, df, p-values and partial eta squared effect sizes) in a table in the main text, and the other ANOVAs in the supplementary materials.

(For large datasets, or papers with a very large number of statistical tests, you may upload a single table file with tests, Ns, etc., with reference to sections in the manuscript.)

**Group allocation**

- Indicate how samples were allocated into experimental groups (in the case of clinical studies, please specify allocation to treatment method); if randomization was used, please also state if restricted randomization was applied
- Indicate if masking was used during group allocation, data collection and/or data analysis

Please outline where this information can be found within the submission (e.g., sections or figure legends), or explain why this information doesn’t apply to your submission:

PD and HC were recruited into their groups based on the presence or absence of a PD diagnosis.

PD patients were pseudorandomly allocated to either be ON or OFF for their first session, restricted to give equal numbers of each.

Patients were aware of which condition they were in, as were experimenters.

This is stated in Methods\Participants

**Additional data files (“source data”)**

- We encourage you to upload relevant additional data files, such as numerical data that are represented as a graph in a figure, or as a summary table
- Where provided, these should be in the most useful format, and they can be uploaded as “Source data” files linked to a main figure or table
- Include model definition files including the full list of parameters used
- Include code used for data analysis (e.g., R, MatLab)
- Avoid stating that data files are “available upon request”

Please indicate the figures or tables for which source data files have been provided:

Source data files are included for all figures, and the figures can be reproduced from these files and the analysis code in the GitHub repo.

Anonymous data is available on OSF, and all Matlab analysis code including figure creation is available on OSF and github.

Links to these are provided in Methods\Data and Code Availability.
